# High early life mortality in free-ranging dogs is largely influenced by humans

**DOI:** 10.1038/srep19641

**Published:** 2016-01-25

**Authors:** Manabi Paul, Sreejani Sen Majumder, Shubhra Sau, Anjan K. Nandi, Anindita Bhadra

**Affiliations:** 1 Department of Biological Sciences, Indian Institute of Science Education and Research Kolkata, India; 2 Department of Physical Sciences, Indian Institute of Science Education and Research Kolkata, India

## Abstract

Free-ranging dogs are a ubiquitous part of human habitations in many developing countries, leading a life of scavengers dependent on human wastes for survival. The effective management of free-ranging dogs calls for understanding of their population dynamics. Life expectancy at birth and early life mortality are important factors that shape life-histories of mammals. We carried out a five year-long census based study in seven locations of West Bengal, India, to understand the pattern of population growth and factors affecting early life mortality in free-ranging dogs. We observed high rates of mortality, with only ~19% of the 364 pups from 95 observed litters surviving till the reproductive age; 63% of total mortality being human influenced. While living near people increases resource availability for dogs, it also has deep adverse impacts on their population growth, making the dog-human relationship on streets highly complex.

Free-ranging dogs (*Canis lupus familiaris*), descendants of pack-living gray wolves (*Canis lupus lupus*), underwent domestication and became a ubiquitous part of human habitations^1^,^2^,^3^,^4^,^5^,^6^. They have managed to survive as scavengers^7^,^8^, co-existing with humans in urban as well as rural habitats. Openly disposed human leftovers, domestic animal carcasses and food received from humans through begging are the major sources of their diet^9^,^10^. While free-ranging dogs depend heavily on humans for sustenance and interact regularly with humans, they are often considered a menace for being carriers of pathogens and zoonosis^11^,^12^,^13^. Sometimes people are attacked by dogs on streets, especially at night when a motor vehicle passes the territory of free-ranging dogs. These free-ranging dogs are often considered as a major threat to human health for being reservoirs of rabies, canine distemper and parvovirus^11^,^12^,^13^. For example, 99% of all human deaths caused by rabies occur in developing countries, with the majority of cases resulting from dog bites^10^. Exponential growth of the human population facilitates population growth of free-ranging dogs^13^. It becomes a serious problem for health management in developing countries where the movement and reproduction of free-ranging dogs are not entirely controlled by humans. Vaccination and animal birth control programmes are expensive, and likely to be effective only if practiced on a large scale^14^,^15^. Improved food waste management could also help to reduce free-ranging dogs in and around human settlements^16^. However in developing countries such management becomes all the more difficult, and management decisions need be founded on an understanding of free-ranging dogs’ ecology and population dynamics, for which scientific data is lacking^17^,^18^.

Free-ranging dogs in India are facultatively social where group dynamics are strongly influenced by social interactions during mating and denning seasons^19^. They have been reported to breed once a year^20^ although two distinct mating seasons have been observed (Sen Majumder and Bhadra, 2015). Mothers, along with other group members show significant amount of cooperative behaviour towards new born pups, which is expected to lead to higher survival rates^21^. Pups start to be weaned around 8 weeks of age, which also marks the onset of conflict over resources with the mother^22^,^23^. The early life of dogs is divided into the pup (0–3 months) and juvenile (3–6 months) stages, and sexual maturity sets in between 6–9 month of age^24^. Dispersals are common in the adult (after attaining sexual maturity) and sub-adult (after 6 months to the attainment of sexual maturity) stages^24^, making the social organization quite dynamic. Although there is a growing body of literature on the behaviour and cognitive abilities of dogs^26^,^27^,^28^,^29^, detailed study of the structure and dynamics of natural free-ranging dog populations are lacking. Life-history comparison of mammals have suggested that juvenile mortality is more highly correlated with life-history traits than adult mortality, and the age of the onset of reproduction in females is strongly correlated with life expectancy at birth^30^,^31^. Since a large number of births are observed in free-ranging dogs every year, but population growth does not appear to be very high^13^,^32^, we speculate that high early life mortality might be an important factor responsible for controlling population growth in free-ranging dogs. In this study we estimate mortality rates in the early life of free-ranging dogs and assess the factors affecting their survival. Since onset of sexual maturity in dogs begins at 6 months of age, pups were tracked from birth to the end of the seventh month of their age or death, whichever was earlier. This ensures that we considered the entire juvenile period, and did not consider sexually mature individuals in estimating early life mortality.

## Results

### Demography

**Figure 1:**
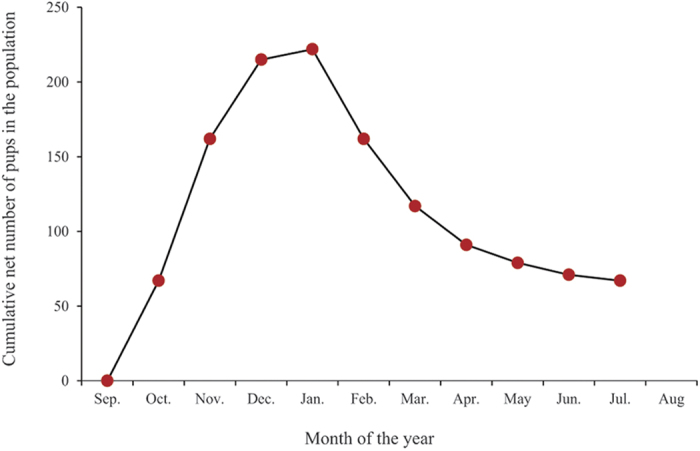
Cumulative net number of pups (births – deaths) observed in each calendar month, for sampling conducted over 5 denning seasons between 2010 and 2015. The population size reached its peak in January after which it steadily declined.

**Figure 2:**
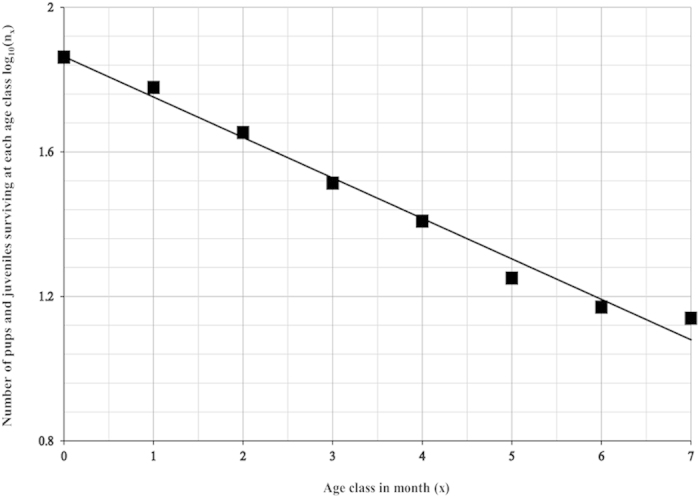
Number of pups and juveniles (data from 2010–2015) that survived at each month of age decreased significantly (on a log_10_ scale) with the increase in age (Linear regression: R^2^ = 0.985, std. β = −0.992, P < 0.0001).

Pups started to appear in the population in October, with the number of pups and juveniles reaching a peak during the months of December and January. The average litter size at birth was 3.98 ± 2 (median 4, quartiles 3–5, N = 108) and there was no bias towards any particular sex (Male: Female: 1:1.04; Mann Whitney U test: U = 5621, df = 104, 104, P = 0.625). The death/disappearance rate overtook the birth rate from January, and the net number of pups and juveniles began to decrease significantly in the population (Linear regression: R^2^ = 0.848, β = −0.921, P = 0.003, Supplementary Information 1), with the number of newborn pups reaching zero by the end of February (Fig. 1). Only 18.96% of total observed pups reached their 7 months of age. There was a negative correlation between dog age and survival (Linear regression: R^2^ = 0.985, β = −0.992, P < 0.0001, Fig. 2) and this trend was comparable over the 5 years of sampling (Linear regression comparison: F = 1.412, P = 0.254). The highest rate of mortality (30.47%) was observed at the 4^th^ month of age.

### Survival analysis

**Figure 3:**
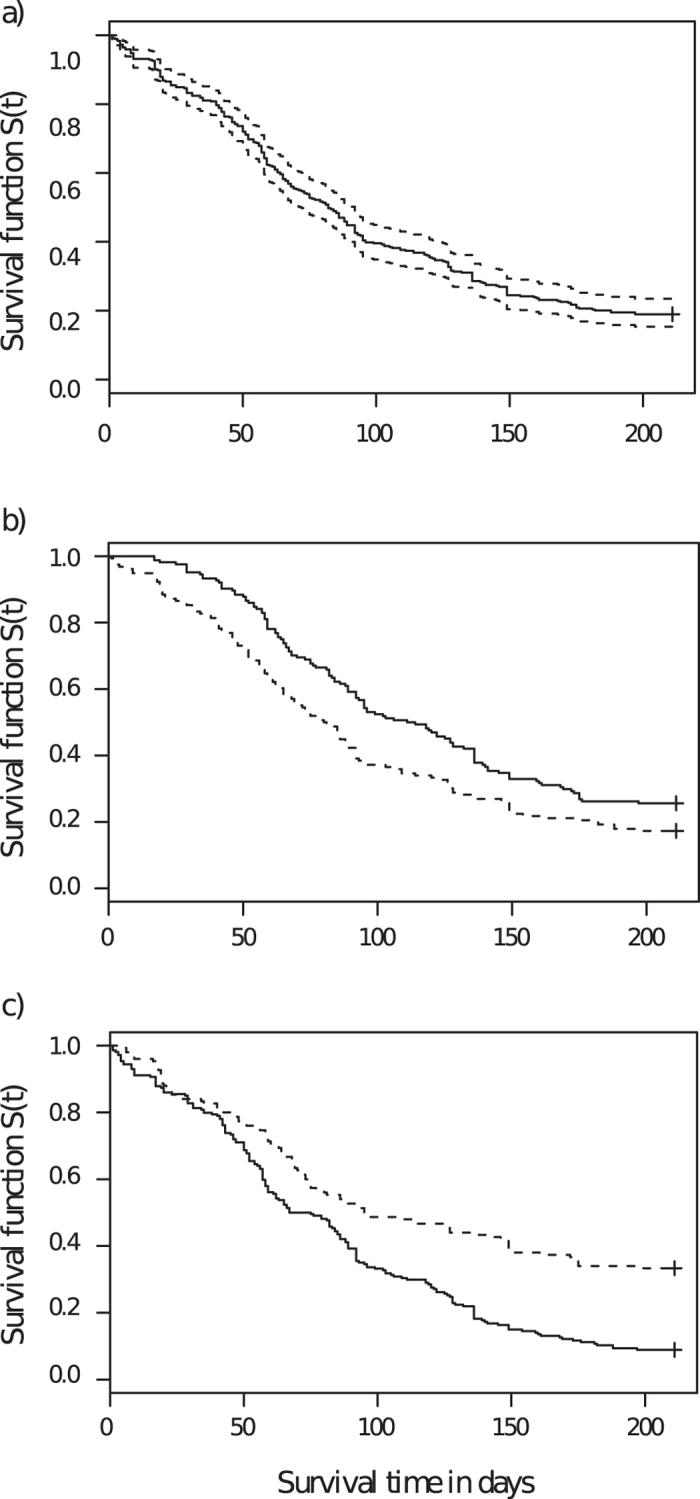
**(a)**A line graph representing the plot of survival probabilities of the pups corresponding to each ordered time at which the event of removal (death) occurred. The median of the curve corresponds to 82 days (N = 364, 95% CL: 72–92 days). The solid line represents the median, and the dashed lines represent the 95% confidence limits. **(b)** Line graph showing the stratified survival curves for males and females separately. The median value for females is 112.5 days and for males is 80.5 days. The solid line represents the survival curve for females while the dashed line is for males. **(c)** Line graph showing the stratified survival curves for urban and suburban habitats separately. The median value of survival time for the urban population is 95 days and for the suburban population is 71 days. Solid line represents the survival curve for suburban and dashed line is for urban habitats.

Survival analysis yielded a plot of survival probabilities of the pups corresponding to each ordered time at which the event of removal occurred (Fig. 3a). The median of the curve corresponds to 82 days (N = 364, 95%CL: 72–92 days). In the Cox mixed-effect model, the variables ‘sex’ and ‘habitat’ showed significant effect on survival time (Supplementary Information 2). Figure 3b shows the stratified survival curves for males and females separately. The median value of survival time for females was 112.5 days (N = 164, 95% CL: 93–136 days) while that for males was much lower, at 80.5 days (N = 156, 95% CL: 68–92 days). A log-rank test confirmed a significant difference (Chisq = 8.1, df = 1, p = 0.0045) between the two curves, showing that the survival probabilities of males were significantly lower than that of females. Figure 3c shows the stratified survival curves for urban and suburban populations separately. The median value of survival time for the urban population was 95 days (N = 150, 95%CL: 75–149 days) while that of the suburban population was 71 days (N = 214, 95%CL: 59–86 days). Log-rank test confirmed a significant difference (Chisq = 26.7, df = 1, p = 2.33e–07) between the two curves. However the survival rates of males and females remained comparable over the two types of habitat (as sex and habitat together had no effect on mortality) (Supplementary Information 2, Model 6).

### Causes of mortality

Only 32% of the total mortality was by natural causes and for 5% of total mortality there were no reliable records. The remaining 63% of the total mortality was influenced directly (accidents/ poisoning/ beatings) or indirectly (taken away from the population) by humans. Natural causes were the prevalent reason for mortality (52%) at age class 0–1 month when only 3% of the mortality was human influenced. From 1 month of pup age human influenced death or disappearance became prevalent (50%) and this trend remained unchanged until the 5^th^ month of age. From the 5^th^ month onwards 70–80% of the total mortality was caused by juveniles going missing (Table 1) which indicated the onset of dispersal from the population.

**Figure 4:**
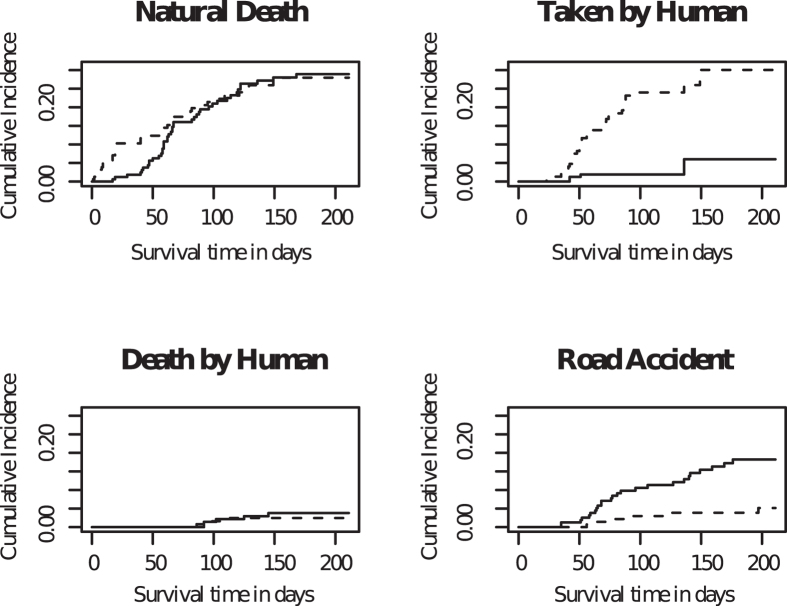
Cumulative incidence curve showing the proportion of individuals removed from the population over time for a specific cause, in the presence of other competing causes. The solid lines represent females, while the dashed lines represent males in the population.

The cumulative incidence curve (Fig. 4) showed the proportion of individuals removed from the population for a specific cause as time passed, in presence of the other competing causes. Males and females showed significantly different incidences for categories 2 (taken by human) and 4 (road accidents) (Table 2; Fig. 4). This suggested that humans preferentially took away male pups and female pups faced more road accidents. Urban and suburban habitats showed significantly different incidences for categories 1 (natural death) and 3 (murdered by human) (Supplementary Information 3), suggesting higher human intervention in suburban habitats.

### Simulation model

**Figure 5:**
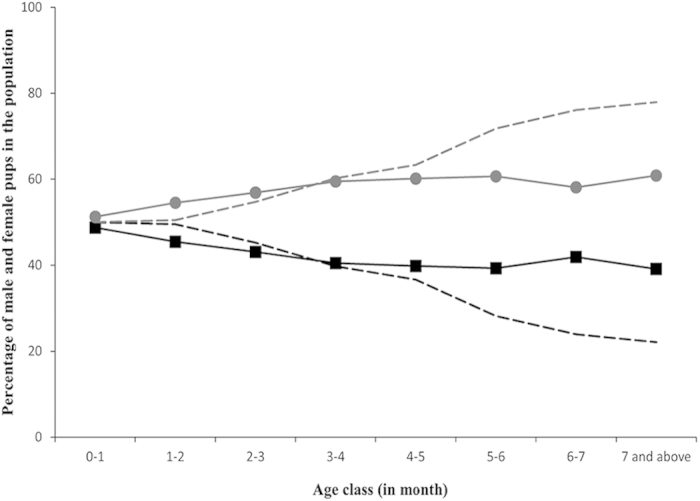
Simulation results for percentage of male and female pups that survived in the population at each month of age. The black lines represent the data for males, gray lines represent data for females, with the solid lines representing real data and the dashed lines representing data from simulations.

The simulation model (Supplementary Information 4) generated a highly skewed population at 7 months, with an expected male:female ratio of 1:3.53, while the real population had a much better sex ratio of 1:1.56. However, the trend for the distribution of sexes at each age class was similar in the model and the data (Fig. 5), suggesting that the observed skew was by chance alone. Thus the higher deaths of females due to road accidents was a consequence of selective removal of male pups from the population, and not an intrinsic tendency of females to be more accident prone.

## Discussion

Early-life mortality in free-ranging dogs was observed to be very high, with only about 19% of the pups reaching the age of sexual maturity, implying an even lower lifetime survival rate. The pattern of mortality over pup age in months did not vary across the four years of sampling, and can thus be considered to be the actual trend in the population of free-ranging dogs. Highest rate of mortality were observed in the 4^th^ month of pup age, which is in agreement with an earlier report of 67% mortality by 4 months^33^. This can be attributed to increased mobility and onset of dispersal in juveniles around the 4^th^ month of age^25^,^34^. Our data also showed an increased incidence of missing individuals after the 5^th^ month of age, suggesting increased dispersal. Studies on other canids that live around human settlements have also suggested higher mortality rates for juveniles, as compared to that of adults^35^,^36^,^37^.

Humans have been identified as a major cause of mortality for many species including coyotes and foxes, that share space with humans, in both rural and urban environments^38^,^39^,^40^,^41^,^42^, and free-ranging dogs are no exception^16^. Most of these studies suggest road kills and/or hunting as human-induced causes of deaths of animals living in human dominated landscapes. Our study too revealed an extremely high incidence (62%) of death or disappearance due to human influenced factors. Interestingly, the human influenced deaths increased and remained high after the first month of age, which is the time at which pups are no longer in the protective environment of dens, and social play increases^43^,^44^. Hence the vulnerability of pups to accidents, and their accessibility to humans who carry them away either for adoption or for a passing fancy increases around this time, leading to increased human influenced mortality, including brutality towards the pups.

The overall higher rates of mortality, both natural and human influenced, observed in urban areas as compared to semi-urban areas is intriguing. Not only were the pups and juveniles more likely to die of natural causes like disease and starvation in the suburbs, where human densities are lower than in cities, but the interference of humans on their lives was also high. The relatively lower engagement of people in the cities with the dogs, and a higher abundance of shelters and resources in cities^16^ probably causes this difference in mortality levels. These results resonate with observations on carnivores such as the red fox (*Vulpes vulpes*), coyote (*Canis latrans*), Eurasian badger (*Meles meles*) and raccoon (*Procyon lotor*) that have not only adapted to urban habitats, but have exploited anthropogenic shelters and food sources to achieve higher population densities than in natural habitats^42^. Thus the dependence of dogs on humans for their survival^16^, and the anthropogenic factors that may influence their population dynamics at later stages of life, like socio-cultural factors^15^, need to be understood in more detail for better management of free-ranging dog populations in urban habitats.

People preferentially remove male pups from the population, and our simulation shows that this leads to higher mortality of females due to accidents, due to the sheer skew in the numbers of male and female pups/juveniles in the resulting population. Thus, humans not only are responsible for a high proportion of the mortality of free-ranging dogs in early life, but also cause a skew in the sex ratio of the cohort that attains sexual maturity. Random sampling from the population of free-ranging dogs of West Bengal have shown that the sex ratio in the dog population does not deviate significantly from 1:1, when both adults and juveniles are considered^32^. Since we observed a skew in the sex ratio at 7 months of age, this suggests that factors like adult mortality and dispersion can lead to stabilization of the sex ratio. Indeed, our simulation suggests that the skew itself can lead to higher mortality of females at a later stage of life due to factors both natural and human-induced. Since life expectancy at birth and juvenile mortality are important factors that shape life-history traits in mammals^30^,^31^, further investigations following our observations might provide important leads for effective and humane management of free-ranging dog populations in India and other countries which face similar issues with free-ranging dogs.

## Methods

### Sampling

Sampling was carried out using a census method (scan sampling by walking on a pre-decided route) on free-ranging dogs between 2010 and 2015 in various parts of West Bengal, India, including urban and semi urban habitats. The study covered the following areas of West Bengal, India: Saltlake (22.5800° N, 88.4200° E), Kalyani (22.9750° N, 88.4344° E), IISER-K campus in Mohanpur (22.9638° N, 88.5246° E), Batanagar (22.29° N, 88.11° E), Garia (22.4598948° N, 88.3894769° E), Howrah (22.59° N, 88.31° E) and Ramnagar, East Midnapore (21.47° N, 87.45° E). The localities included residential as well as commercial areas, and were selected randomly based on convenience of sampling within human habitations. Each of those localities had a study area of 1–2 km^2^. The observer visited the pre-selected area at least twice or if possible thrice a day at random times and walked on all roads and lanes to locate pups. Each visit took 1–2 hours of time depending on the size of the study area. Whenever pups were sighted, a record was made including details like the location of the litter, litter size, sex of the pups and their date of birth (approximate, when actual date of birth was not known). These details were used later to track individual pups. 95 of these litters (having a total of 364 pups) were followed up to the age of 7 months. For each of the cases of pup death (or disappearance) we recorded the causes of death either from direct observations or relying on reports by the local people. We carried out linear regression analyses in StatixtiXL version 1.11 to understand the pattern of mortality in the population and for comparing the data from the different years.

### Demography

108 mother-litter groups of free-ranging dogs were located between 2010 and 2015, of which 95 litters comprising of 364 pups in total were tracked till the 7^th^ month of age, while for the remaining 13 litters (66 pups) only information at birth was available. Tracking relied on the coat colour and patch positions on their body. All deaths and disappearances were considered under the category of mortality. The various factors that caused death could be broadly divided into natural causes (disease, climatic factors, predation by other dogs or jackals, and injury from fights), human influenced deaths (poisoning, beating, malnutrition, road accidents, taken away from the population) and disappearances for unknown reasons. Pups are often taken away by humans (mostly children), and our qualitative observations show that while some of these pups are adopted as pets, most are abandoned. The abandoned pups mostly die of starvation, unless they are further adopted by other people. Abandoned juveniles typically face aggression from adult dogs if they are not released back in their own groups, and they too rarely survive. Hence pups and juveniles taken by humans were considered to be dead for this analysis as these were effectively removed from the gene pool, unless we saw them return to their natal groups within the span of our observations.

### Survival analysis

In the survival analysis the survival function *S(t)* is defined by the probability that an individual survives longer than some specified time t. In our case, ‘survival’ means survival in the population, so all the individuals that were removed from the population by various causes observed were considered as ‘not-survived’. The ‘survival time’ means the age in days of the individual at which the ‘event’ of his/her removal occurred. The event of death/removal is also typically referred as a ‘failure’. Since the individuals were followed until 30 weeks of their age, and no information about the survival time is available after that period, the survival time of the individuals are considered as ‘right-censored’. In order to understand the influence of the various factors in shaping the population structure of free-ranging dogs, we carried out a survival analysis^45^,^46^ by the Kaplan-Meir (KM) method, using the ‘Survival’ package^47^,^48^ of the statistical computation software R^49^. ‘Survfit’ function of ‘KM method’ generates the survival curve considering the survival function *S(t)* as the dependent variable while survival time was considered as the independent variable (Fig. 3a). KM survival estimates for males and females and urban and suburban populations separately (Fig. 3b,c). In order to test the effect of explanatory variables; litter size, sex and habitat, on the survival time of the individuals, we used the Cox proportional hazard (PH) model^50^. The Cox modified chi-square statistic was used to compare differences in type of death for each sex with the help of ‘coxme’ package^51^ of R’ (For details, see Supplementary Note 2) using the raw data (For details, see Supplementary Note 5).

### Causes of mortality

We analyzed the effect of all types of causes (natural death, human influenced death, disappearance or gone missing from the population and death or disappearance due to unknown reasons) behind pups’ mortality, for each age class (0–1 month, 1–2, 2–3 and so on till 7 month of pup age). Later we stratified the ‘human influenced death’ category into three subcategories such as the death or disappearance records for ‘taken by humans’ and ‘murdered by humans’ and ‘death in road accident’.

We used competing risk analysis to understand the contribution of various factors to mortality in the population. We divided the event of “death” into various categories. Therefore, considering the fact that more than one event is possible, the individual can experience only one of the several different types of events over the study period. These categorical events are 1) ‘natural death’, 2) ‘taken by human’, 3) ‘murdered by human’, 4) ‘death in road accident’ and 5) ‘no information’ (includes ‘missing’ and ‘unknown’) (Table 2). We used a cumulative incidence curve (CIC) to model competing risks survival data using the ‘cmprsk’ package^52^ of R (Fig. 4). CIC provides estimates of the marginal probability of an event in the presence of competing events. A modified chi-square statistic^53^, for differences in incidence among the different sexes was used for comparisons.

### Simulation model

We were intrigued by the observation that a higher percentage of females died due to road accidents, and wanted to test if females are intrinsically more prone to road accidents, or this observation was the result of a skew caused by selective removal of males. We carried out a simulation using the observed mortality rates applied to an initial population of 1000 dogs having a sex ratio of 1:1. Two kinds of mortality rates were used in the model- taken by humans and others.

### Ethical statement

No dogs were harmed during this work. All work reported here was purely observation based, and did not involve handling of dogs in any manner. The methods reported in this paper were approved by the animal ethics committee of IISER Kolkata (approval number: 1385/ac/10/CPCSEA), and in accordance with approved guidelines of animal rights regulations of the Government of India.

## Additional Information

**How to cite this article**: Paul, M. *et al*. High early life mortality in free-ranging dogs is largely influenced by humans. *Sci. Rep*. **6**, 19641; doi: 10.1038/srep19641 (2016).

## Supplementary Material

Supplementary Information

## Figures and Tables

**Table 1:** Percentage of pups that died at each age class due to four mortality types; human influenced death, gone missing from the population, natural death and unknown reasons.

Age class (Age of pup in month)	Human influenced death	Gone missing from the population	Natural death	Unknown
0–1	3.1	37.5	**51.6**	7.8
1–2	**49.3**	17.3	26.7	6.7
2–3	**38.7**	22.6	33.9	4.8
3–4	25.7	**37.1**	31.4	5.7
4–5	**46.2**	30.8	20.5	2.6
5–6	20	**73.3**	6.7	0
6–7	20	**80**	0	0

Bolded data shows the highest death records for each age class.

**Table 2:** Raw data and results of the competing risk analysis.

Raw data						
**Category**	**0**	**1**	**2**	**3**	**4**	**5**
**Female**	42	42	8	5	**25**	42
**Male**	27	38	**39**	3	6	43
**Results**						
**Category**	**Statistic**			**P**		**df**
1	0.004			9.48e–01		1
2	26.24			**3.02e–07**		1
3	0.41			5.27e–01		1
4	11.81			**5.91e–04**		1
5	0.25			6.15e–01		1

Death was divided into five categories; 1) natural death, 2) taken by human, 3) murdered by human, 4) death in road accident and 5) no information.
