# Supplementary material for: High early life mortality in free-ranging dogs is largely influenced by humans
Source: Sci Rep. 2016 Jan 25;6:19641. doi: 10.1038/srep19641 (PMC4726281; doi:10.1038/srep19641)
Supplement: Supplementary Information [file srep19641-s1.pdf]

High early life mortality in free-ranging dogs is largely influenced by humans

Manabi Paul[1], Sreejani Sen Majumder[1], Shubhra Sau[1], Anjan K. Nandi[2] and Anindita Bhadra[1*]

Supplementary Information

[1] Department of Biological Sciences,
Indian Institute of Science Education and Research Kolkata, India

[2] Department of Physical Sciences,
Indian Institute of Science Education and Research Kolkata, India

[*]**Address for Correspondence:**

Behaviour and Ecology Lab, Department of Biological Sciences,
Indian Institute of Science Education and Research Kolkata
Mohanpur Campus, Mohanpur,
PIN 741246, West Bengal, INDIA
tel. 91-33-25873119 *fax* +91-33-25873020 *e-mail:* abhadra@iiserkol.ac.in

Supplementary Information 1

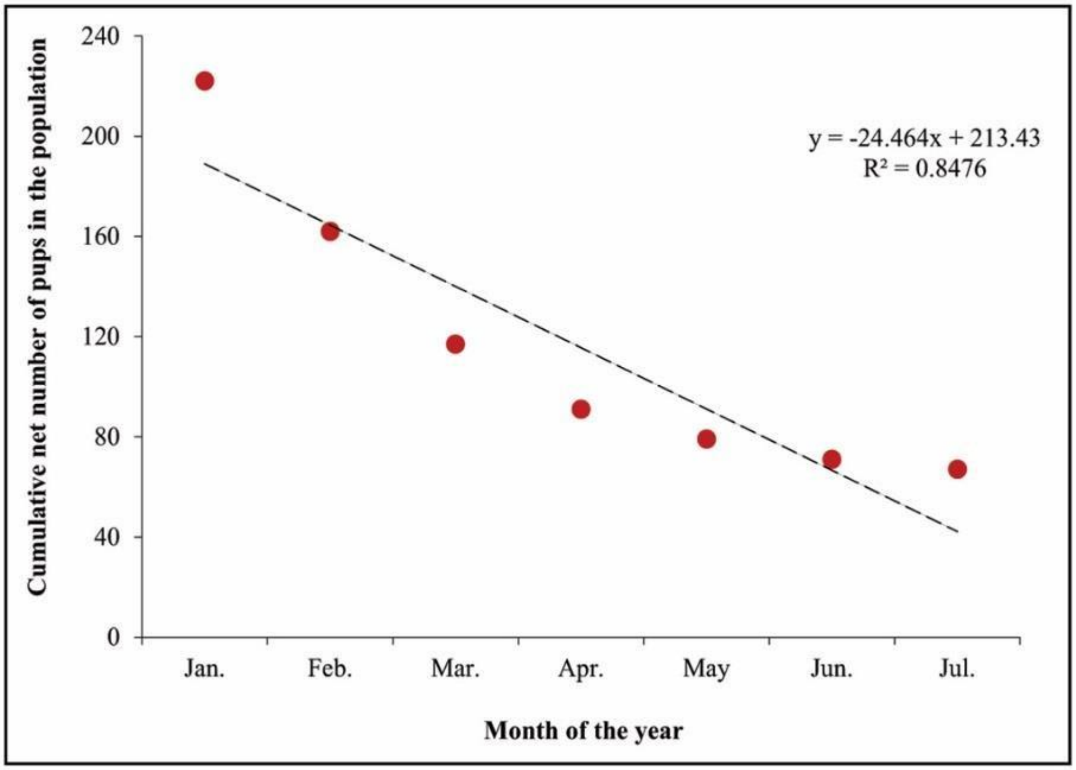

Supplementary Figure 1: Line graph showing the significant decrease in the cumulative net number of pups in the population (Linear regression: $R^2 = 0.848$, std. $\beta = -0.921$, $P = 0.003$) from the month of January.

Supplementary Information 2

Details of the Cox proportional hazard (PH) model

The used variables are:

Litter size- **Ls**

Sex of the individual pup- **fsx** (Male- **M**, Female- **F**)

Habitat types- **fhb** (Urban- **UR**, Suburban- **SU**)

Year of data collection- **fyr**

Litter identity- **flt**

We started with the full model:

Model 1 <- coxme(Surv(Age,Srv)~Ls*fsx*fhb+(1|fyr/flt))

Fixed coefficients

	**coef**	**exp(coef)**	**se(coef)**	**z**	**p**
Ls	0.01570455	1.0158285	0.07715722	0.20	0.8400
fsxM	0.84094228	2.3185507	0.48797975	1.72	0.0850
fhbUR	-1.43324636	0.2385333	0.53548240	-2.68	**0.0074**
Ls:fsxM	-0.09957380	0.9052231	0.09994753	-1.00	0.3200
Ls:fhbUR	0.09531852	1.1000092	0.09495200	1.00	0.3200
fsxM:fhbUR	0.30250525	1.3532448	0.65003978	0.47	0.6400
Ls:fsxM:fhbUR	0.01859136	1.0187653	0.11831918	0.16	0.8800

Random effects

Group	Variable	Std Dev	Variance
fyr/flt	(Intercept)	0.29959233	0.08975556
fyr	(Intercept)	0.27205404	0.07401340

AIC- 2570.593 BIC- 2656.297

Since the three-way interaction is not significant, we have dropped the term and run the model again.

Model 2: Surv(Age, Srv) ~ Ls * fsx + Ls * fhb + fsx * fhb + (1 | fyr/flt)

Fixed coefficients

	**coef**	**exp(coef)**	**se(coef)**	**z**	**p**
Ls	0.008998816	1.0090394	0.06416382	0.14	0.8900
fsxM	0.779890845	2.1812342	0.29713058	2.62	**0.0087**
fhbUR	-1.483858605	0.2267610	0.42607622	-3.48	**0.0005**
Ls:fsxM	-0.086267492	0.9173488	0.05319201	-1.62	0.1000
Ls:fhbUR	0.104993312	1.1107032	0.07196555	1.46	0.1400
fsxM:fhbUR	0.394229189	1.4832405	0.28747741	1.37	0.1700

Random effects

Group	Variable	Std Dev	Variance
fyr/flt	(Intercept)	0.29896284	0.08937878
fyr	(Intercept)	0.27207534	0.07402499

We have compared our recent model with the previous one and the result represents no difference between these two models. Model comparisons justify the dropping of the three-way term.

anova(Model 1,Model 2)

Analysis of Deviance Table

Cox model: response is Surv(Age, Srv)

Model 1: ~Ls * fsx * fhb + (1 | fyr/flt)

Model 2: ~Ls * fsx + Ls * fhb + fsx * fhb + (1 | fyr/flt)

<table>
<tr><th></th><th>loglik</th><th>Chisq</th><th>Df</th><th>P(>|Chi|)</th></tr>
<tr><td>1</td><td>-1282.2</td><td></td><td></td><td></td></tr>
<tr><td>2</td><td>-1282.2</td><td>0.0255</td><td>1</td><td>0.8731</td></tr>
</table>

	AIC	BIC
**Model 1**	2570.593	2656.297
**Model 2**	2568.74	2650.93

Since the last three two-way interactions (Ls:fsxM, Ls:fhbUR, fsxM:fhbUR) have no significant effect on the survival of the pups, we have dropped those terms one by one and run the model (please see Model 3 to Model 7) and have compared them with Model 2.

Model 3: Surv(Age, Srv) ~ Ls * fsx + fsx * fhb + (1 | fyr/flt)

Fixed coefficients

	**coef**	**exp(coef)**	**se(coef)**	**z**	**p**
Ls	0.07372565	1.0765114	0.04698772	1.57	1.2e-01
fsxM	0.78701974	2.1968395	0.30807567	2.55	**1.1e-02**
fhbUR	-0.96819539	0.3797678	0.23222708	-4.17	**3.1e-05**
Ls:fsxM	-0.08268252	0.9206434	0.05453325	-1.52	1.3e-01
fsxM:fhbUR	0.34832693	1.4166953	0.28312017	1.23	2.2e-01

Random effects

**Group**	**Variable**	**Std Dev**	**Variance**
fyr/flt	(Intercept)	0.31669814	0.10029771
fyr	(Intercept)	0.26152076	0.06839311

anova (Model 2,**Model 3**)

Model 2: ~Ls * fsx + Ls * fhb + fsx * fhb + (1 | fyr/flt)

Model 3: ~Ls * fsx + fsx * fhb + (1 | fyr/flt)

	loglik	Chisq	Df	P(>|Chi|)
1	-1282.2			
2	-1283.2	2.0782	1	**0.1494**

	AIC	BIC
**Model 2**	2568.74	2650.93
**Model 3**	2569.095	2653.573

Model 4: Surv(Age, Srv) ~ Ls * fhb + fsx * fhb + (1 | fyr/flt)

Fixed coefficients

	coef	exp(coef)	se(coef)	z	p
Ls	-0.03471206	0.9658835	0.05779131	-0.60	0.55000
fhbUR	-1.37040645	0.2540037	0.40691242	-3.37	**0.00076**
fsxM	0.38180764	1.4649303	0.16814933	2.27	**0.02300**
Ls:fhbUR	0.09520048	1.0998793	0.07106405	1.34	0.18000
fhbUR:fsxM	0.29710307	1.3459540	0.27756514	1.07	0.28000

Random effects

Group	Variable	Std Dev	Variance
fyr/flt	(Intercept)	0.29156310	0.08500904
fyr	(Intercept)	0.26369259	0.06953378

anova(Model 2,**Model 4**)

Model 2: ~Ls * fsx + Ls * fhb + fsx * fhb + (1 | fyr/flt)

Model 4: ~Ls * fhb + fsx * fhb + (1 | fyr/flt)

<table>
<tr><th></th><th>loglik</th><th>Chisq</th><th>Df</th><th>P(>|Chi|)</th></tr>
<tr><td>1</td><td>-1282.2</td><td></td><td></td><td></td></tr>
<tr><td>2</td><td>-1283.5</td><td>2.6155</td><td>1</td><td>0.1058</td></tr>
</table>

	AIC	BIC
**Model 2**	2568.74	2650.93
**Model 4**	2569.801	2646.503

Model 5: Surv(Age, Srv) ~ Ls * fsx + Ls * fhb + (1 | fyr/flt)

Fixed coefficients

	coef	exp(coef)	se(coef)	z	p
Ls	0.004841435	1.0048532	0.06526170	0.07	0.9400
fsxM	0.850131724	2.3399551	0.29287713	2.90	**0.0037**
fhbUR	-1.223097327	0.2943172	0.37821047	-3.23	**0.0012**
Ls:fsxM	-0.070718539	0.9317241	0.05184056	-1.36	0.1700
Ls:fhbUR	0.095917285	1.1006680	0.07254510	1.32	0.1900

Random effects

Group	Variable	Std Dev	Varianc	
fyr/flt	(Intercept)	0.31310339	0.09803373	fyr
	(Intercept)	0.26550228	0.07049146	

anova (Model 2,**Model 5**)

Model 2: ~Ls * fsx + Ls * fhb + fsx * fhb + (1 | fyr/flt)

Model 5: ~Ls * fsx + Ls * fhb + (1 | fyr/flt)

loglik	Chisq	Df	P(>|Chi|)

1 -1282.2			
2 -1283.1	1.9027	1	**0.1678**

	AIC	BIC			
**Model 2**	2568.74	2650.93	**Model 5**	2568.349	2650.918

--

Model 6: Surv(Age, Srv) ~ Ls + fhb * fsx + (1 | fyr/flt)

Fixed coefficients

	**coef**	**exp(coef)**	**se(coef)**	**z**	**p**
Ls	0.02617522	1.0265208	0.03558231	0.74	4.6e-01
fhbUR	-0.91849163	0.3991206	0.22668846	-4.05	**5.1e-05**
fsxM	0.39377100	1.4825610	0.16821560	2.34	**1.9e-02**
fhbUR:fsxM	0.28426561	1.3287858	0.27811186	1.02	3.1e-01

Random effects

**Group**	**Variable**	**Std Dev**	**Variance**
fyr/flt	(Intercept)	0.30561067	0.09339788
fyr	(Intercept)	0.25462376	0.06483326

anova (Model 2,**Model 6**)

Model 2: ~Ls * fsx + Ls * fhb + fsx * fhb + (1 | fyr/flt)

Model 6: ~Ls + fhb * fsx + (1 | fyr/flt)

	**loglik**	**Chisq**	**Df**	**P(>|Chi|)**
1	-1282.2			
2	-1284.4	4.3705	2	**0.1124**

	**AIC**	**BIC**
**Model 2**	2568.74	2650.93
**Model 6**	2569.932	2647.916

Model 7: Surv (Age, Srv) ~ Ls + fhb + fsx + (1 | fyr/flt)

Fixed coefficients

	**coef**	**exp(coef)**	**se(coef)**	**z**	**p**

Ls 0.02612448 1.0264687 0.03600451 0.73 4.7e-01 fhbUR -0.76601451 0.4648621 0.16541716 -4.63 **3.6e-06** fsxM 0.50049802 1.6495426 0.13346754 3.75 **1.8e-04**

Random effects

Group	Variable	Std Dev	Variance
fyr/flt	(Intercept)	0.31801808	0.10113550
fyr	(Intercept)	0.24977424	0.06238717

anova(Model 2,**Model 7**)

Model 2: ~Ls * fsx + Ls * fhb + fsx * fhb + (1 | fyr/flt)

Model 7: ~Ls + fhb + fsx + (1 | fyr/flt)

	loglik	Chisq	Df	P(>|Chi|)
1	-1282.2			
2	-1284.9	5.4174	3	**0.1437**

	AIC	BIC
**Model 2**	2568.74	2650.93
**Model 7**	2568.76	2646.738

Supplementary Information 3

Cumulative Incidence Curve for Habitats

Survived- **0**

Natural death-**1**

Taken by human- **2**

Murdered by human- **3**

Death in road accident- **4**

No information about their death but disappeared from the population- **5**

The frequency distribution for the different habitats is as follows:

Category	**0**	**1**	**2**	**3**	**4**	**5**
Suburban (SU)	19	**70**	27	**16**	18	64
Urban (UR)	50	24	20	0	13	43

Disappearance due to natural causes and death by human are significantly higher in suburban population in compare to the urban population.

**Category**	**stat**	**p**	**df**

1	11.10519852	**0.0008608607**	1
2	0.01882155	0.8908792995	1
3	11.67067374	**0.0006349301**	1
4	0.02139737	0.8837016160	1
5	0.18833068	0.6643096252	1

Supplementary 4

Simulation model for mortality in dog populations

Variables used:

Total number of male pups survived- **m**

Total number of female pups survived- **f**

Number of male pups died per 100 pups- **mdp**

Number of female pups died per 100 pups- **fdp**

The details of the program mortality

```
<- function(m,f,mdp,tdp){

  # Total number of male and female at the beginning
m_init <- m   f_init <- f

  # Male death percentage
  MDP <- mdp/100

  # Total death percentage
  TDP <- tdp/100
```

```
  # Number of male alive (after removing dead males)
m_dead <- m_init*MDP   m_alive <- m_init -
m_dead

  # Total number of male and females alive
tot_alive <- m_alive + f_init

  # Total number of individual dies
tot_death <- tot_alive*TDP

  # Creating blank lists for putting 1000 iteration values
m_res <- numeric(1000)   f_res <- numeric(1000)

  # Iterating mortality in one age class 1000 times
for (j in 1:1000){     list <- c("M", "F")     death
<- numeric(tot_death)     for (i in 1:tot_death){
death[i] <- sample(list, 1)
    }
    m_res[j] = m_alive - length(subset(death, death == "M"))   # Dead males
f_res[j] = f_init - length(subset(death, death == "F"))  #Dead females
  }
```

```
  # Average of 1000 iterations of mortality in one age class
m_res_avg <- mean(m_res) # Males   f_res_avg <-
mean(f_res) # Females

  # Final result   result <- c("Males Alive=", m_res_avg, "Females
Alive=", f_res_avg)   return(result)
}
```

Supplementary 5

Raw data showing details of the 95 litters used for the analysis of pup survival up to 7th month of age.

Year of data collection	Mother's identity	Litter size	Pup's identity	Sex	Age in days	Survived till 7 month of pup age*	Cause of death	Habitat type
2010-2011	M1	7	P1	M	136	1	Being taken by human	Suburban
2010-2011	M1	7	P2	M	136	1	Being taken by human	Suburban
2010-2011	M1	7	P3	F	136	1	Being taken by human	Suburban
2010-2011	M1	7	P4	F	136	1	Being taken by human	Suburban
2010-2011	M1	7	P5	F	136	1	Being taken by human	Suburban
2010-2011	M1	7	P6	F	136	1	Being taken by human	Suburban
2010-2011	M1	7	P7	F	136	1	Being taken by human	Suburban
2010-2011	M2	4	P8	F	211	0		Suburban
2010-2011	M2	4	P9	M	211	0		Suburban
2010-2011	M2	4	P10	F	149	1	Disease	Suburban
2010-2011	M2	4	P11	M	126	1	Inter group fight	Suburban
2010-2011	M3	8	P12	M	128	1	Missing	Suburban
2010-2011	M3	8	P13	M	128	1	Missing	Suburban
2010-2011	M3	8	P14	F	128	1	Missing	Suburban
2010-2011	M3	8	P15	F	128	1	Missing	Suburban
2010-2011	M3	8	P16	F	149	1	Missing	Suburban
2010-2011	M3	8	P17	M	188	1	Missing	Suburban
2010-2011	M3	8	P18	M	182	1	Missing	Suburban

2010-2011	M3	8	P19	F	169	1	Road accident	Suburban
2010-2011	M4	2	P20	M	129	1	Disease	Suburban
2010-2011	M4	2	P21	F	211	0		Suburban
2010-2011	M5	4	P22	M	92	1	Poison	Suburban
2010-2011	M5	4	P23	F	92	1	Poison	Suburban
2010-2011	M5	4	P24	F	92	1	Poison	Suburban
2010-2011	M5	4	P25	F	211	0		Suburban
2010-2011	M6	1	P26	M	121	1	Disease	Suburban
2010-2011	M7	4	P27	U	57	1	Poison	Suburban
2010-2011	M7	4	P28	U	57	1	Poison	Suburban
2010-2011	M7	4	P29	U	57	1	Poison	Suburban
2010-2011	M7	4	P30	U	57	1	Poison	Suburban
2010-2011	M8	3	P31	M	67	1	Disease	Suburban
2010-2011	M8	3	P32	F	67	1	Disease	Suburban
2010-2011	M8	3	P33	F	67	1	Disease	Suburban
2010-2011	M9	4	P34	U	50	1	Poison	Suburban
2010-2011	M9	4	P35	U	50	1	Poison	Suburban
2010-2011	M9	4	P36	U	50	1	Poison	Suburban
2010-2011	M9	4	P37	U	50	1	Poison	Suburban
2010-2011	M10	5	P38	M	79	1	Missing	Suburban
2010-2011	M10	5	P39	F	96	1	Road accident	Suburban
2010-2011	M10	5	P40	F	127	1	Road accident	Suburban
2010-2011	M10	5	P41	F	122	1	Inter group fight	Suburban
2010-2011	M10	5	P42	F	160	1	Missing	Suburban

2010-2011	M11	4	P43	M	92	1	Missing	Suburban
2010-2011	M11	4	P44	M	92	1	Missing	Suburban
2010-2011	M11	4	P45	M	92	1	Missing	Suburban
2010-2011	M11	4	P46	F	92	1	Missing	Suburban
2010-2011	M12	3	P47	M	181	1	Missing	Suburban
2010-2011	M12	3	P48	F	82	1	Missing	Suburban
2010-2011	M12	3	P49	F	82	1	Missing	Suburban
2010-2011	M13	4	P50	F	29	1	Missing	Suburban
2010-2011	M13	4	P51	F	29	1	Missing	Suburban
2010-2011	M13	4	P52	F	29	1	Missing	Suburban
2010-2011	M13	4	P53	M	154	1	Missing	Suburban
2010-2011	M14	5	P54	F	125	1	Malnutrition	Suburban
2010-2011	M14	5	P55	F	145	1	Malnutrition	Suburban
2010-2011	M14	5	P56	F	159	1	Road accident	Suburban
2010-2011	M14	5	P57	F	161	1	Missing	Suburban
2010-2011	M14	5	P58	M	45	1	Being taken by human	Suburban
2010-2011	M15	13	P59	F	95	1	Missing	Urban
2010-2011	M15	13	P60	F	95	1	Missing	Urban
2010-2011	M15	13	P61	F	95	1	Missing	Urban
2010-2011	M15	13	P62	F	95	1	Missing	Urban
2010-2011	M15	13	P63	F	175	1	Missing	Urban
2010-2011	M15	13	P64	F	175	1	Missing	Urban
2010-2011	M15	13	P65	F	175	1	Missing	Urban
2010-2011	M15	13	P66	M	149	1	Disease	Urban

2010-2011	M15	13	P67	M	149	1	Disease	Urban
2010-2011	M15	13	P68	M	149	1	Being taken by human	Urban
2010-2011	M15	13	P69	M	149	1	Being taken by human	Urban
2010-2011	M15	13	P70	M	149	1	Being taken by human	Urban
2010-2011	M15	13	P71	M	149	1	Being taken by human	Urban
2010-2011	M16	3	P72	M	86	1	Being taken by human	Urban
2010-2011	M16	3	P73	M	86	1	Being taken by human	Urban
2010-2011	M16	3	P74	F	141	1	Missing	Urban
2010-2011	M17	4	P75	F	114	1	Disease	Urban
2010-2011	M17	4	P76	F	211	0		Urban
2010-2011	M17	4	P77	F	197	1	Missing	Urban
2010-2011	M17	4	P78	M	211	0		Urban
2010-2011	M18	3	P79	M	54	1	Unknown	Urban
2010-2011	M18	3	P80	F	173	1	Missing	Urban
2010-2011	M18	3	P81	F	211	0		Urban
2010-2011	M19	4	P82	M	65	1	Missing	Urban
2010-2011	M19	4	P83	M	161	1	Missing	Urban
2010-2011	M19	4	P84	F	211	0		Urban
2010-2011	M19	4	P85	F	211	0		Urban
2010-2011	M20	4	P86	M	80	1	Missing	Urban
2010-2011	M20	4	P87	M	127	1	Missing	Urban
2010-2011	M20	4	P88	F	127	1	Unknown	Urban
2010-2011	M20	4	P89	M	127	1	Missing	Urban
2010-2011	M21	5	P90	M	60	1	Unknown	Urban

2010-2011	M21	5	P91	M	211	0		Urban
2010-2011	M21	5	P92	M	109	1	Missing	Urban
2010-2011	M21	5	P93	F	73	1	Missing	Urban
2010-2011	M21	5	P94	F	211	0		Urban
2011-2012	M22	3	P95	M	188	1	Missing	Suburban
2011-2012	M22	3	P96	M	211	0		Suburban
2011-2012	M22	3	P97	F	211	0		Suburban
2011-2012	M23	3	P98	M	65	1	Cold	Suburban
2011-2012	M23	3	P99	F	65	1	Cold	Suburban
2011-2012	M23	3	P100	F	211	0		Suburban
2011-2012	M24	5	P101	U	43	1	Missing	Suburban
2011-2012	M24	5	P102	U	43	1	Missing	Suburban
2011-2012	M24	5	P103	U	43	1	Missing	Suburban
2011-2012	M24	5	P104	U	43	1	Missing	Suburban
2011-2012	M24	5	P105	U	43	1	Missing	Suburban
2011-2012	M25	7	P106	M	82	1	Stomach infection	Suburban
2011-2012	M25	7	P107	F	141	1	Road accident	Suburban
2011-2012	M25	7	P108	F	120	1	Inter group fight	Suburban
2011-2012	M25	7	P109	F	63	1	Cold	Suburban
2011-2012	M25	7	P110	F	66	1	Inter group fight	Suburban
2011-2012	M25	7	P111	F	66	1	Inter group fight	Suburban
2011-2012	M25	7	P112	F	29	1	Disease	Suburban
2011-2012	M26	5	P113	M	85	1	Missing	Suburban
2011-2012	M26	5	P114	F	139	1	Missing	Suburban

2011-2012	M26	5	P115	M	173	1	Missing	Suburban
2011-2012	M26	5	P116	F	119	1	Inter group fight	Suburban
2011-2012	M26	5	P117	F	211	0		Suburban
2011-2012	M27	6	P118	M	211	0		Suburban
2011-2012	M27	6	P119	U	5	1	Suffocation	Suburban
2011-2012	M27	6	P120	U	5	1	Suffocation	Suburban
2011-2012	M27	6	P121	U	8	1	Suffocation	Suburban
2011-2012	M27	6	P122	U	8	1	Suffocation	Suburban
2011-2012	M27	6	P123	U	8	1	Suffocation	Suburban
2011-2012	M28	5	P124	F	99	1	Inter group fight	Suburban
2011-2012	M28	5	P125	M	75	1	Worm infection	Suburban
2011-2012	M28	5	P126	F	96	1	Disease	Suburban
2011-2012	M28	5	P127	F	168	1	Disease	Suburban
2011-2012	M28	5	P128	M	105	1	Beaten up by human	Suburban
2011-2012	M29	3	P129	M	2	1	Suffocation	Suburban
2011-2012	M29	3	P130	M	41	1	Being taken by human	Suburban
2011-2012	M29	3	P131	M	41	1	Being taken by human	Suburban
2011-2012	M30	4	P132	U	19	1	Missing	Suburban
2011-2012	M30	4	P133	U	20	1	Missing	Suburban
2011-2012	M30	4	P134	U	20	1	Missing	Suburban
2011-2012	M30	4	P135	U	20	1	Missing	Suburban
2011-2012	M31	3	P136	U	15	1	Unknown	Suburban
2011-2012	M31	3	P137	F	122	1	Disease	Suburban
2011-2012	M31	3	P138	F	149	1	Road accident	Suburban

2011-2012	M32	5	P139	M	88	1	Disease	Suburban
2011-2012	M32	5	P140	M	38	1	Being taken by human	Suburban
2011-2012	M32	5	P141	F	47	1	Worm infection	Suburban
2011-2012	M32	5	P142	F	40	1	Worm infection	Suburban
2011-2012	M32	5	P143	F	106	1	Road accident	Suburban
2011-2012	M33	2	P144	M	28	1	Being taken by human	Suburban
2011-2012	M33	2	P145	F	211	0		Suburban
2011-2012	M34	4	P146	F	59	1	Disease	Suburban
2011-2012	M34	4	P147	M	59	1	Disease	Suburban
2011-2012	M34	4	P148	F	59	1	Disease	Suburban
2011-2012	M34	4	P149	M	56	1	Road accident	Suburban
2011-2012	M35	1	P150	U	57	1	Missing	Suburban
2011-2012	M36	6	P151	M	56	1	Being taken by human	Suburban
2011-2012	M36	6	P152	M	52	1	Missing	Suburban
2011-2012	M36	6	P153	F	118	1	Missing	Suburban
2011-2012	M36	6	P154	F	84	1	Road accident	Suburban
2011-2012	M36	6	P155	F	211	0		Suburban
2011-2012	M36	6	P156	M	86	1	Poison	Suburban
2011-2012	M37	3	P157	F	57	1	Missing	Suburban
2011-2012	M37	3	P158	F	89	1	Disease	Suburban
2011-2012	M37	3	P159	M	34	1	Missing	Suburban
2011-2012	M38	2	P160	M	86	1	Being taken by human	Suburban
2011-2012	M38	2	P161	M	29	1	Missing	Suburban
2011-2012	M39	4	P162	F	62	1	Inter group fight	Suburban

2011-2012	M39	4	P163	F	82	1	Natural	Suburban
2011-2012	M39	4	P164	M	4	1	Suffocation	Suburban
2011-2012	M39	4	P165	M	3	1	Suffocation	Suburban
2011-2012	M40	4	P166	M	71	1	Missing	Urban
2011-2012	M40	4	P167	M	211	0		Urban
2011-2012	M40	4	P168	F	136	1	Road accident	Urban
2011-2012	M40	4	P169	M	94	1	Being taken by human	Urban
2011-2012	M41	2	P170	M	211	0		Urban
2011-2012	M41	2	P171	M	146	1	Missing	Urban
2011-2012	M42	7	P172	F	172	1	Missing	Urban
2011-2012	M42	7	P173	F	211	0		Urban
2011-2012	M42	7	P174	F	211	0		Urban
2011-2012	M42	7	P175	M	73	1	Road accident	Urban
2011-2012	M42	7	P176	M	68	1	Being taken by human	Urban
2011-2012	M42	7	P177	M	211	0		Urban
2011-2012	M42	7	P178	F	68	1	Road accident	Urban
2011-2012	M43	5	P179	F	211	0		Urban
2011-2012	M43	5	P180	F	64	1	Road accident	Urban
2011-2012	M43	5	P181	M	211	0		Urban
2011-2012	M43	5	P182	F	70	1	Unknown	Urban
2011-2012	M43	5	P183	M	211	0		Urban
2011-2012	M44	3	P184	F	211	0		Urban
2011-2012	M44	3	P185	M	211	0		Urban
2011-2012	M44	3	P186	M	88	1	Being taken by human	Urban

2011-2012	M45	1	P187	M	211	0		Urban
2011-2012	M46	6	P188	U	16	1	Disease	Urban
2011-2012	M46	6	P189	M	211	0		Urban
2011-2012	M46	6	P190	M	114	1	Unknown	Urban
2011-2012	M46	6	P191	F	211	0		Urban
2011-2012	M46	6	P192	F	211	0		Urban
2011-2012	M46	6	P193	M	46	1	Being taken by human	Urban
2011-2012	M47	3	P194	M	211	0		Urban
2011-2012	M47	3	P195	F	68	1	Road accident	Urban
2011-2012	M47	3	P196	F	211	0		Urban
2011-2012	M48	4	P197	M	211	0		Urban
2011-2012	M48	4	P198	M	69	1	Being taken by human	Urban
2011-2012	M48	4	P199	M	211	0		Urban
2011-2012	M48	4	P200	M	127	1	Missing	Urban
2012-2013	M49	6	P201	M	58	1	Inter group fight	Suburban
2012-2013	M49	6	P202	M	58	1	Inter group fight	Suburban
2012-2013	M49	6	P203	M	62	1	Inter group fight	Suburban
2012-2013	M49	6	P204	F	58	1	Inter group fight	Suburban
2012-2013	M49	6	P205	F	42	1	Inter group fight	Suburban
2012-2013	M49	6	P206	F	211	0		Suburban
2012-2013	M50	3	P207	U	4	1	Missing	Suburban
2012-2013	M50	3	P208	U	4	1	Missing	Suburban
2012-2013	M50	3	P209	U	4	1	Missing	Suburban
2012-2013	M51	6	P210	M	51	1	Being taken by human	Suburban

2012-2013	M51	6	P211	M	92	1	Missing	Suburban
2012-2013	M51	6	P212	F	52	1	Road accident	Suburban
2012-2013	M51	6	P213	F	89	1	Missing	Suburban
2012-2013	M51	6	P214	F	51	1	Being taken by human	Suburban
2012-2013	M51	6	P215	F	111	1	Inter group fight	Suburban
2012-2013	M52	2	P216	F	86	1	Disease	Suburban
2012-2013	M52	2	P217	F	50	1	Disease	Suburban
2012-2013	M53	2	P218	M	3	1	Inter group fight	Suburban
2012-2013	M53	2	P219	M	197	1	Road accident	Suburban
2012-2013	M54	1	P220	U	1	1	At birth	Suburban
2012-2013	M55	4	P221	U	9	1	Missing	Suburban
2012-2013	M55	4	P222	U	9	1	Missing	Suburban
2012-2013	M55	4	P223	U	9	1	Missing	Suburban
2012-2013	M55	4	P224	U	9	1	Missing	Suburban
2012-2013	M56	6	P225	U	17	1	Missing	Suburban
2012-2013	M56	6	P226	U	17	1	Missing	Suburban
2012-2013	M56	6	P227	U	17	1	Missing	Suburban
2012-2013	M56	6	P228	F	17	1	Missing	Suburban
2012-2013	M56	6	P229	F	140	1	Road accident	Suburban
2012-2013	M56	6	P230	F	136	1	Stomach infection	Suburban
2012-2013	M57	5	P231	M	120	1	Road accident	Suburban
2012-2013	M57	5	P232	M	211	0		Suburban
2012-2013	M57	5	P233	F	176	1	Road accident	Suburban
2012-2013	M57	5	P234	F	211	0		Suburban

2012-2013	M57	5	P235	F	211	0		Suburban
2012-2013	M58	3	P236	M	31	1	Missing	Suburban
2012-2013	M58	3	P237	M	31	1	Missing	Suburban
2012-2013	M58	3	P238	M	31	1	Missing	Suburban
2012-2013	M59	4	P239	M	52	1	Being taken by human	Suburban
2012-2013	M59	4	P240	M	52	1	Being taken by human	Suburban
2012-2013	M59	4	P241	F	211	0		Suburban
2012-2013	M59	4	P242	F	59	1	Stomach infection	Suburban
2012-2013	M60	4	P243	M	65	1	Disease	Suburban
2012-2013	M60	4	P244	F	62	1	Disease	Suburban
2012-2013	M60	4	P245	F	58	1	Disease	Suburban
2012-2013	M60	4	P246	F	211	0		Suburban
2012-2013	M61	4	P247	M	42	1	Being taken by human	Suburban
2012-2013	M61	4	P248	F	42	1	Being taken by human	Suburban
2012-2013	M61	4	P249	F	42	1	Being taken by human	Suburban
2012-2013	M61	4	P250	F	211	0		Suburban
2012-2013	M62	3	P251	M	46	1	Disease	Suburban
2012-2013	M62	3	P252	F	46	1	Disease	Suburban
2012-2013	M62	3	P253	F	57	1	Disease	Suburban
2012-2013	M63	3	P254	M	17	1	Disease	Suburban
2012-2013	M63	3	P255	U	17	1	Disease	Suburban
2012-2013	M63	3	P256	F	84	1	Disease	Suburban
2012-2013	M64	3	P257	M	23	1	Being taken by human	Suburban
2012-2013	M64	3	P258	F	132	1	Missing	Suburban

2012-2013	M64	3	P259	F	118	1	Missing	Suburban
2012-2013	M65	2	P260	M	18	1	Unknown	Urban
2012-2013	M65	2	P261	F	211	0		Urban
2012-2013	M66	7	P262	M	19	1	Disease	Urban
2012-2013	M66	7	P263	M	19	1	Disease	Urban
2012-2013	M66	7	P264	M	19	1	Disease	Urban
2012-2013	M66	7	P265	M	19	1	Disease	Urban
2012-2013	M66	7	P266	F	19	1	Disease	Urban
2012-2013	M66	7	P267	F	211	0		Urban
2012-2013	M66	7	P268	F	211	0		Urban
2012-2013	M67	6	P269	M	58	1	Being taken by human	Urban
2012-2013	M67	6	P270	M	20	1	Unknown	Urban
2012-2013	M67	6	P271	M	211	0		Urban
2012-2013	M67	6	P272	F	34	1	Unknown	Urban
2012-2013	M67	6	P273	F	51	1	Road accident	Urban
2012-2013	M67	6	P274	F	211	0		Urban
2012-2013	M68	2	P275	F	63	1	Road accident	Urban
2012-2013	M68	2	P276	F	211	0		Urban
2012-2013	M69	5	P277	M	17	1	Disease	Urban
2012-2013	M69	5	P278	M	35	1	Being taken by human	Urban
2012-2013	M69	5	P279	M	211	0		Urban
2012-2013	M69	5	P280	M	17	1	Disease	Urban
2012-2013	M69	5	P281	F	17	1	Disease	Urban
2012-2013	M70	3	P282	M	59	1	Road accident	Urban

2012-2013	M70	3	P283	M	50	1	Being taken by human	Urban
2012-2013	M70	3	P284	F	211	0		Urban
2012-2013	M71	1	P285	M	211	0		Urban
2012-2013	M72	3	P286	M	211	0		Urban
2012-2013	M72	3	P287	F	59	1	Unknown	Urban
2012-2013	M72	3	P288	F	211	0		Urban
2012-2013	M73	5	P289	M	9	1	Inter group fight	Urban
2012-2013	M73	5	P290	M	9	1	Inter group fight	Urban
2012-2013	M73	5	P291	M	211	0		Urban
2012-2013	M73	5	P292	M	19	1	Disease	Urban
2012-2013	M73	5	P293	F	211	0		Urban
2012-2013	M74	3	P294	M	25	1	Missing	Urban
2012-2013	M74	3	P295	F	25	1	Missing	Urban
2012-2013	M74	3	P296	F	211	0		Urban
2012-2013	M75	5	P297	M	74	1	Being taken by human	Urban
2012-2013	M75	5	P298	M	88	1	Being taken by human	Urban
2012-2013	M75	5	P299	M	211	0		Urban
2012-2013	M75	5	P300	F	211	0		Urban
2012-2013	M75	5	P301	F	81	1	Road accident	Urban
2013-2014	M76	4	P302	M	48	1	Unknown	Urban
2013-2014	M76	4	P303	M	64	1	Missing	Urban
2013-2014	M76	4	P304	M	211	0		Urban
2013-2014	M76	4	P305	F	75	1	Missing	Urban
2013-2014	M77	3	P306	U	23	1	Disease	Urban

2013-2014	M77	3	P307	U	23	1	Disease	Urban
2013-2014	M77	3	P308	U	23	1	Disease	Urban
2013-2014	M78	5	P309	M	46	1	Being taken by human	Urban
2013-2014	M78	5	P310	M	63	1	Missing	Urban
2013-2014	M78	5	P311	F	211	0		Urban
2013-2014	M78	5	P312	F	77	1	Road accident	Urban
2013-2014	M78	5	P313	F	211	0		Urban
2013-2014	M79	2	P314	M	56	1	Missing	Urban
2013-2014	M79	2	P315	M	93	1	Unknown	Urban
2013-2014	M80	3	P316	M	75	1	Unknown	Urban
2013-2014	M80	3	P317	F	211	0		Urban
2013-2014	M80	3	P318	F	65	1	Road accident	Urban
2013-2014	M81	2	P319	U	6	1	Natural	Urban
2013-2014	M81	2	P320	U	6	1	Natural	Urban
2013-2014	M82	4	P321	M	8	1	Disease	Urban
2013-2014	M82	4	P322	M	48	1	Being taken by human	Urban
2013-2014	M82	4	P323	M	211	0		Urban
2013-2014	M82	4	P324	F	41	1	Inter group fight	Urban
2013-2014	M83	3	P325	M	40	1	Missing	Urban
2013-2014	M83	3	P326	F	75	1	Unknown	Urban
2013-2014	M83	3	P327	F	211	0		Urban
2013-2014	M84	2	P328	M	40	1	Disease	Urban
2013-2014	M84	2	P329	M	40	1	Disease	Urban
2013-2014	M85	3	P330	M	21	1	Unknown	Urban

2013-2014	M85	3	P331	M	211	0		Urban
2013-2014	M85	3	P332	F	59	1	Road accident	Urban
2013-2014	M86	4	P333	M	69	1	Being taken by human	Urban
2013-2014	M86	4	P334	M	72	1	Being taken by human	Urban
2013-2014	M86	4	P335	F	58	1	Road accident	Urban
2013-2014	M86	4	P336	U	5	1	Unknown	Urban
2013-2014	M87	2	P337	F	211	0		Urban
2013-2014	M87	2	P338	F	211	0		Urban
2014-2015	M88	5	P339	M	95	1	Disease	Suburban
2014-2015	M88	5	P340	M	85	1	Being taken by human	Suburban
2014-2015	M88	5	P341	M	109	1	Natural	Suburban
2014-2015	M88	5	P342	F	88	1	Inter group fight	Suburban
2014-2015	M88	5	P343	M	103	1	Inter group fight	Suburban
2014-2015	M89	4	P344	M	79	1	Being taken by human	Suburban
2014-2015	M89	4	P345	F	93	1	Missing	Suburban
2014-2015	M89	4	P346	F	76	1	Road accident	Suburban
2014-2015	M89	4	P347	F	89	1	Missing	Suburban
2014-2015	M90	2	P348	M	81	1	Inter group fight	Suburban
2014-2015	M90	2	P349	F	103	1	Disease	Suburban
2014-2015	M91	2	P350	M	1	1	At birth	Suburban
2014-2015	M91	2	P351	U	1	1	At birth	Suburban
2014-2015	M92	2	P352	F	83	1	Missing	Suburban
2014-2015	M92	2	P353	F	102	1	Poison	Suburban
2014-2015	M93	4	P354	M	89	1	Road accident	Suburban

2014-2015	M93	4	P355	F	35	1	Road accident	Suburban
2014-2015	M93	4	P356	F	35	1	Road accident	Suburban
2014-2015	M93	4	P357	F	55	1	Worm infection	Suburban
2014-2015	M94	5	P358	M	46	1	Being taken by human	Suburban
2014-2015	M94	5	P359	M	61	1	Being taken by human	Suburban
2014-2015	M94	5	P360	F	54	1	Missing	Suburban
2014-2015	M94	5	P361	F	54	1	Missing	Suburban
2014-2015	M94	5	P362	F	47	1	Worm infection	Suburban
2014-2015	M95	2	P363	M	52	1	Being taken by human	Suburban
2014-2015	M95	2	P364	M	211	0		Suburban

*Survived till 7 month of pup age- 0, Died before 7 month of pup age- 1
